# Supplementary material for: Micro-RNAs in Response to Active Forms of Vitamin D3 in Human Leukemia and Lymphoma Cells
Source: Int J Mol Sci. 2022 Apr 30;23(9):5019. doi: 10.3390/ijms23095019 (PMC9104187; doi:10.3390/ijms23095019)
Supplement: Supplementary file 1 [file ijms-23-05019-s001.zip › ijms-1652269-SI.pdf]

Suplementary materials

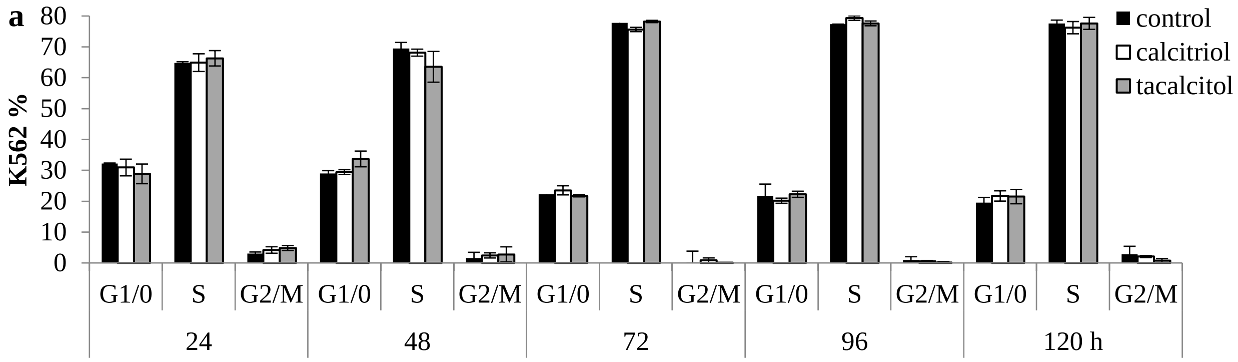

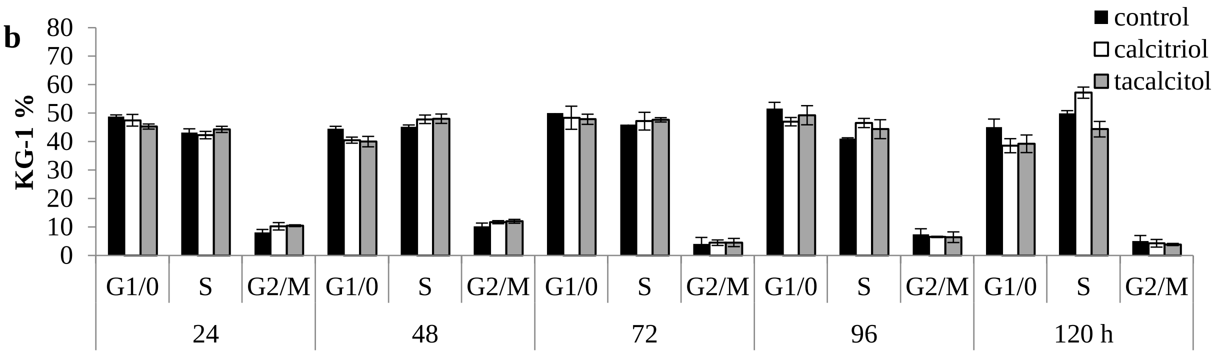

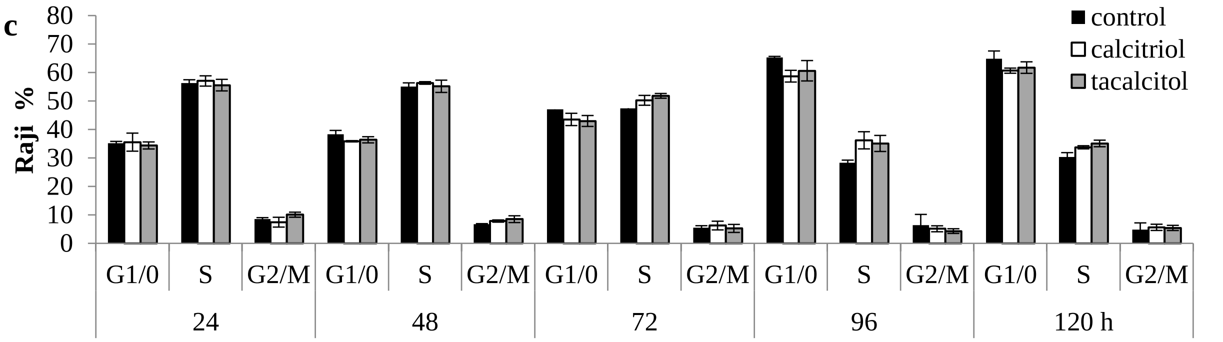

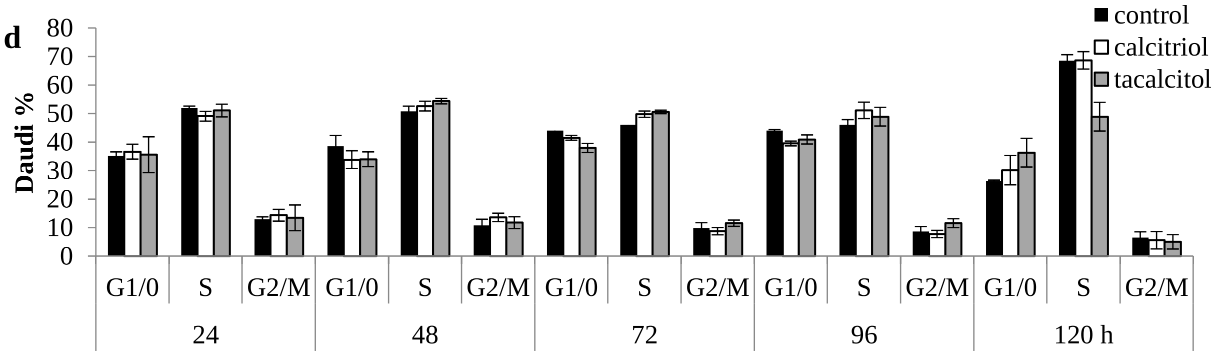

e

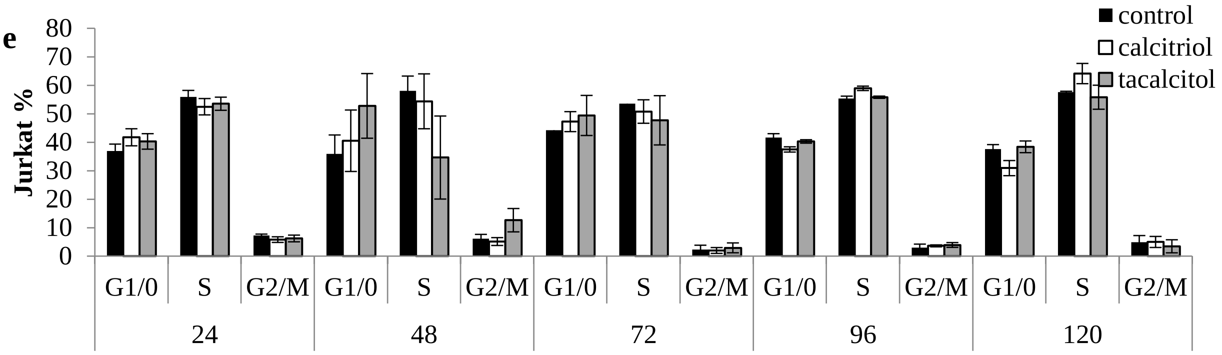

f

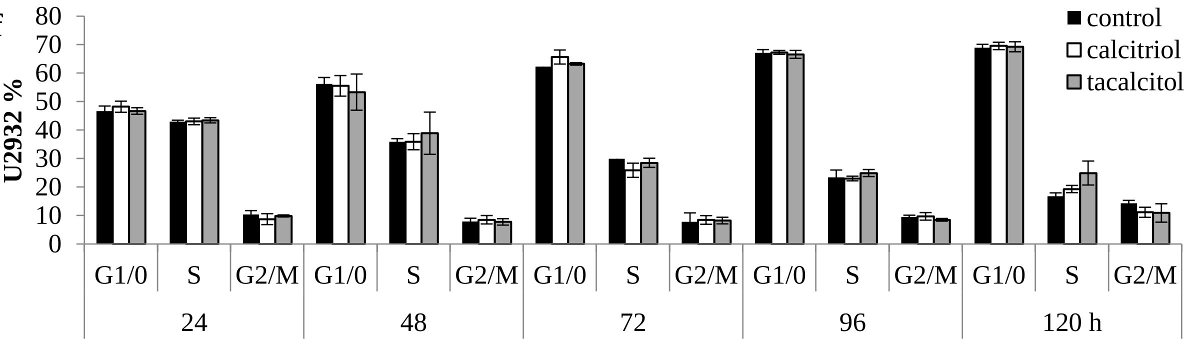

Figure S1. Cell cycle analysis of leukemia: chronic myeloid leukemia K562 (a), acute myeloid leukemia KG-1 (b) and lymphoma cells Burkitt Raji lymphoma (c), Burkitt Daudi lymphoma (d), Jurkat T cell acute leukemia (e), and diffuse large B cell lymphoma U2932 (f) after calcitriol and tacalcitol. Cell cycle analysis after every 24 h after incubation with a single 10 nM dose of calcitriol and tacalcitol.

a

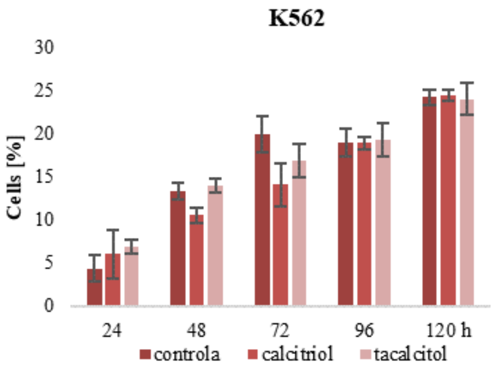

b

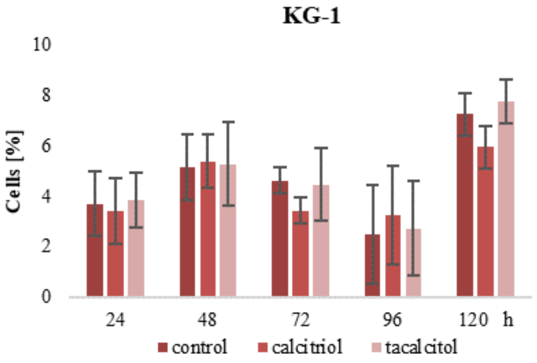

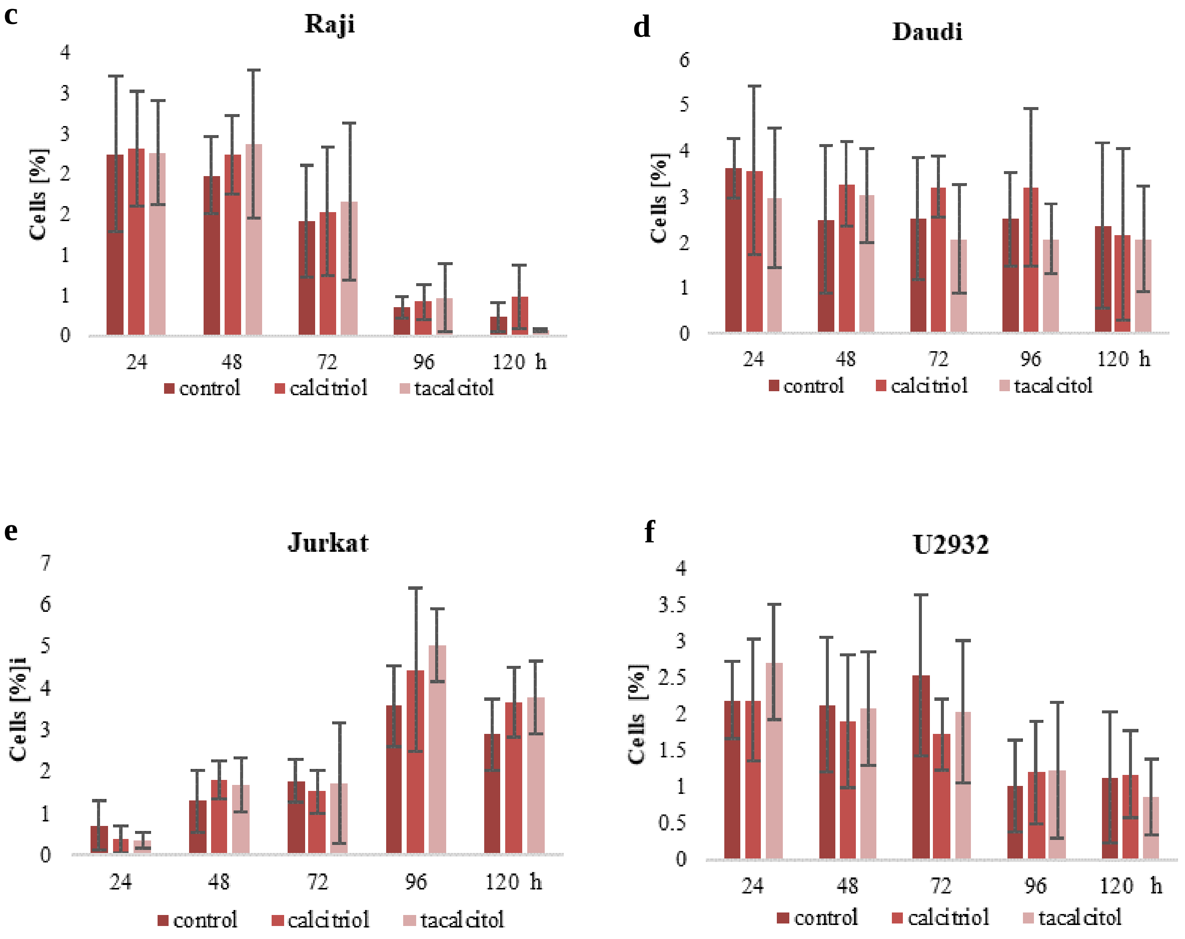

Figure S2. Aneuploid cells in leukemia and lymphoma cella. Calcitriol and tacalcitol treatment did not impact the percentage of aneuploid cells in K562 (a), KG-1 (b), Raji (c), Daudi (d), Jurkat (e), and U2932 (f) cells. The graph shows the mean ± standard deviation of three independent experiments.

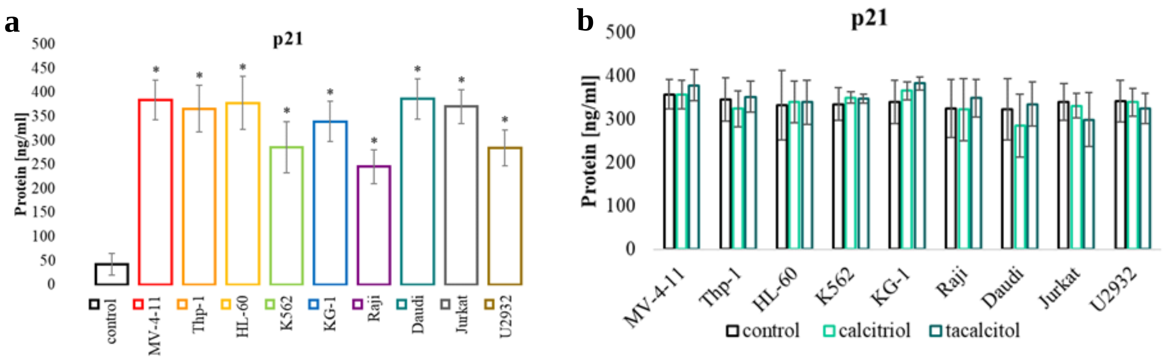

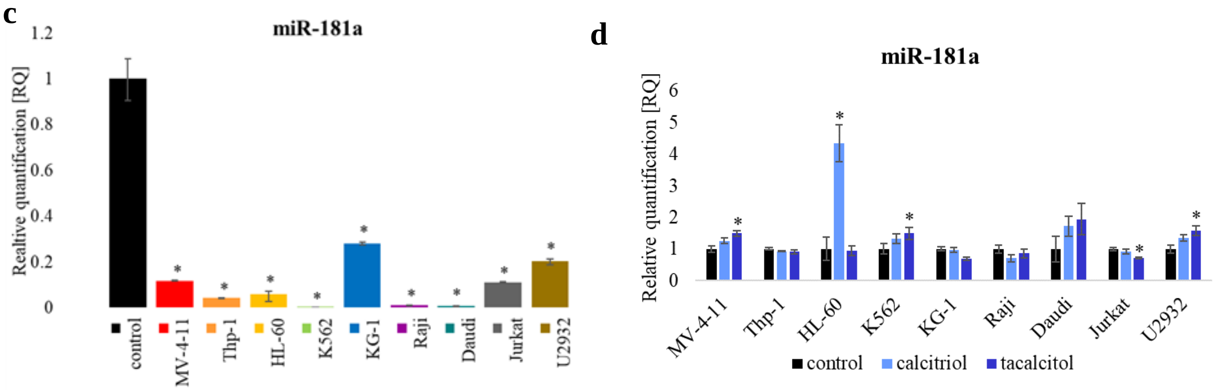

Figure S3. P21 (a and b) and miR-181a (c and d) in human leukemia and lymphoma cells compared to the control: (a) and (c) PBMCs; (b) and (d) untreated cells of the appropriate cell line. Relative quantification (RQ) was calculated using as control: (a) and (c) PBMCs; (b) and (d) untreated cells of the appropriate cell line. * - statistical significance (p <0.05) compared to the control.

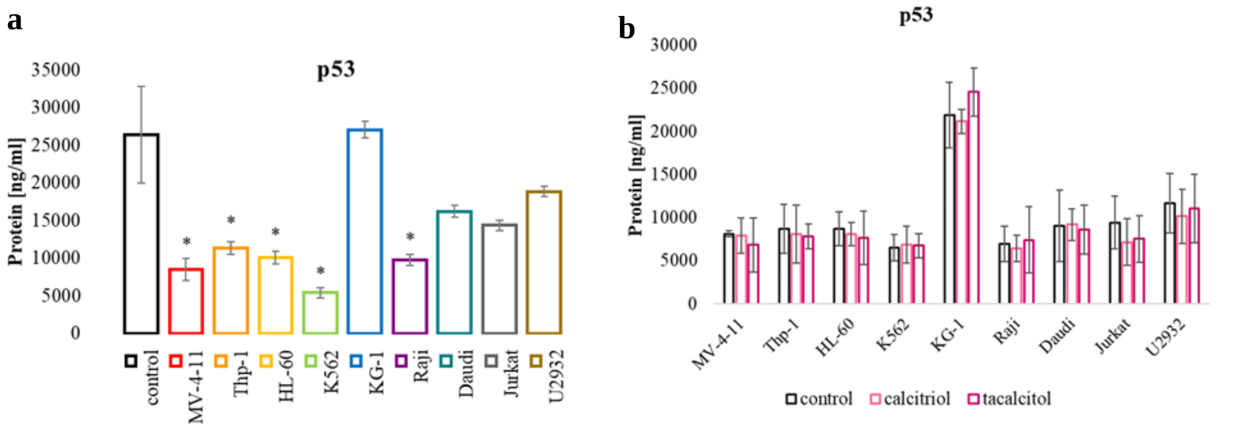

Figure S4. p53 in human leukemia and lymphoma cells compared the control: (a) PBMCs; (b) untreated cells of the appropriate cell line. Relative quantification (RQ) was calculated using as control: (a) PBMCs; (b) untreated cells of the appropriate cell line. The graph shows the mean ± standard deviation. * - statistical significance (p <0.05).

Based on the data from the COSMIC database, the Catalog Of Somatic Mutations In Cancer, whether mutations in the p53 protein are present in leukemia and lymphoma cells that were selected for the study. Interestingly, it turns out that two of the sensitive cell lines, MV-4-11, and HL-60, have the wild-type p53 protein, while the mutant form of the p53 protein is present in the remaining cell lines (Table S1).

Table S1. p53 mutation status in human leukemia and lymphoma cells.

	MV-4-11	*Thp-1*	*HL-60*	*K562*	*KG-1*	*Raji*	*Daudi*	*Jurkat*	*U2932*
p53	WT	MUT	WT	MUT	MUT	MUT	MUT	MUT	MUT
